# Ovariotomy, Its Propriety

**Published:** 1853-07

**Authors:** 


### SELECTIONS

FROM

### FOREIGN AND HOME MEDICAL JOURNALS.

---

*Ovariotomy, its propriety.*—Without in the least degree coming forward as advocates of the operation, but considering that it has been, and still is, occasionally sanctioned and performed by men of ability and great knowledge, and that we read of successful as well as unsuccessful cases, we do think it would have been more to the purpose if Dr. Lee had entered fully into the question, and more effective than a sweeping expression of opinion. We will try to supply Dr. Lee's omission very briefly.

The objections to the operation adduced by Dr. Lee are—1, the great mortality, which, according to his tables, is 1 in $2\frac{18}{42}$; 2, the extreme difficulty of diagnosis, so as to be sure that the case is one which will offer no obstacles to the removal of the tumour; 3, the possibility of prolonging life considerably by other means.

To this it is answered, by the advocates of the operation:

1. Undoubtedly the mortality is very great—1 in $\frac{18}{42}$ according to Dr. Lee, 1 in 3 according to others; but a mortality nearly, if not quite, as great, is not considered a fatal objection to other operations. If we take the major amputations of the limbs (primary and secondary) it appears that in Paris, according to Malgaigne, the mortality is upwards of 1 in 2; in Glasgow, it is 1 in $2\frac{1}{2}$; in British hospitals, it is 1 in $3\frac{1}{2}$. As to amputation of the thigh, Mr. Syme observes—"the stern evidence of hospital statistics shows, that the average frequency of death is not less than from 60 to 70 per cent." Of 987 cases collected by Mr. Phillips, 435 proved fatal, or 44 per cent. Mr. Curling states—"On refering to a table of amputations in the hospitals of London, performed from 1837 to 1843, I find 134 cases of amputation of the thigh and leg, of which 55 were fatal, giving a mortality of 41 per cent." Of 201 amputations of the thigh performed in the Parisian hospitals, and reported by Malgaigne, 126 ended fatally. In the Edinburgh hospitals, 21 died out of 43. Even if we take much larger numbers, we find the mortality very high. Mr. Inman has collected 3586 cases of "amputations generally, primary and secondary, for accident or disease, and the deaths are 1 in $3\frac{1}{10}$." In 4937, published by Mr. Fenwick, the mortality is 1 in $3\frac{1}{15}$.

The result of amputation at the hip joint is still more unfavorable. Mr. Sands Cox has shown, that of 84 cases, 26 were successful, and 58 unsuccessful.

Again, take the operation for hernia. Sir A. Cooper records 36 deaths in 77 operations; and Dr. Inman, 260 deaths in 545 cases. Or, the ligature of large arteries, of which Mr. Phillips has collected 171 cases, of which 57 died; Dr. Inman, 199 cases, of which 66 died. Of 40 cases of ligature of the subclavian artery, 18 proved fatal. Ligature of the innominata has, we believe, been fatal in every case.

So that, taking the mortality at Dr. Lee's estimate, it is not higher than that of other operations, which are admitted to be justifiable notwithstanding.

But, although these figures show that as high a mortality occurs in other operations as in ovariotomy, we beg to remark, that the necessity for the operation is much more urgent in the former: in many cases it is the alternative of immediate death. Further, the operation of ovariotomy is of two kinds—by the long and short incision; and the advocates of the latter point to their statistics, which give a mortality of 4 in 23 cases, or nearly 1 in 6; whilst, according to Mr. Safford Lee's tables, that by the long incision is 1 in 3.

2. The errors in diagnosis have been very great, and the fair inference therefrom is, that the diagnosis is difficult and obscure. But, unless it can be proved that all improvement in this department is impossible, it is clear the argument cuts both ways. If the present deficient diagnosis entails an increased mortality, it is certain that every improvement will by so much reduce it. And we can see that it is possible that this may occur, for if all who have operated had the means of adequately ascertaining the actual presence of a tumor, of being sure that it is an ovarian, of determining the amount of adhesions, and had been sufficiently attentive to the constitution of the patient, it is clear that many of the recorded operations would never have been undertaken, and equally clear that many of the deaths would have been avoided, as a cursory glance at Dr. Lee's tables will prove. Moreover, it seems highly probable that a more accurate knowledge of the contents of these cysts may lead to important results as to the selection of the more promising cases for the operation, which may yet further diminish the mortality; and lastly, it is quite possible, that some beneficial modification of the mode of operating might be adopted.

3. With regard to the prolongation of life by palliative treatment and repeated tapping, it is not easy to estimate the exact gain; it would have been a valuable argument if Dr. Lee had given us a collection of cases to show the amount of prolonged life thus obtained. If the patient be otherwise in good health, and the ovarian tumor increase very slowly, it is true that years may elapse, under careful treatment, without much distress, or any necessity for measures involving risk. In such cases life will be best prolonged by letting the patient alone. But with those that increase rapidly, and to such an extent as to occasion inconvenience and distress, or to threaten life, something must be done to afford relief, and tapping has been the ordinary resource. We have, however, but few statistics to show the results. The only statement of the kind within our reach at this moment is one by Mr. Southam, who, in twenty cases, found that "fourteen died within nine months of the first operation, four of whom survived it only a few days. Of the remaining six, two died in eighteen months, and four lived for periods varying from four to nearly nine years. It further appears that paracentesis does not prolong life, on an average, for more than eighteen months and nineteen days, and that one in four dies from the effect of the first operation." Undoubtedly, these numbers are too small to enable us to form a correct judgment; but, so far as they go, they do not advocate very strongly the operation of tapping.

From this brief summary it appears that the admissibility of the operation will depend not so much upon the rate of mortality hitherto, as upon future improvements in diagnosis; and when we see men of high intelligence like Drs. Simpson and Bennet, Southam, Walne, and Frederick Bird, devoting themselves to this task, we cannot doubt that a decided and practical advance will be made.

Having laid these facts before the profession, we shall leave it to decide how far Dr. Lee's condemnation is just. —*Brit. and Foreign Medico-Chirurgical Review.*

